# Curcumin Inhibits The Adverse Effects of Sodium Arsenite in Mouse Epididymal Sperm 

**DOI:** 10.22074/ijfs.2016.4916

**Published:** 2016-06-01

**Authors:** Hamid Reza Momeni, Najmeh Eskandari

**Affiliations:** Department of Biology, Faculty of Science, Arak University, Arak, Iran

**Keywords:** Curcumin, Natural Antioxidant, Sodium Arsenite

## Abstract

**Background:**

The aim of this study was to investigate the effects of curcumin on epididy- mal sperm parameters in adult male Navel Medical Research Institute (NMRI) mice ex- posed to sodium arsenite.

**Materials and Methods:**

In this experimental study, we divided the animals into four groups: control, sodium arsenite (5 mg/kg), curcumin (100 mg/kg) and curcumin+sodium arsenite. Exposures were performed by intraperitoneal injections for a 5-week period. After the exposure period, we recorded the animals’ body and left testes weights. The left caudal epididymis was used to count the sperm number and analyze motility, viability, morphological abnormalities, acrosome reaction, DNA integrity, and histone-protamine replacement in the spermatozoa. One-way analysis of variance (ANOVA) followed by the Tukey’s test was used to assess the statistical significance of the data with SPSS 16.0. P<0.05 was considered significant.

**Results:**

Mice exposed to sodium arsenite showed a significant decrease in the num- ber, motility, viability, normal sperm morphology and acrosome integrity of spermato- zoa compared to the control group. In the curcumin+sodium arsenite group, curcumin significantly reversed these adverse effects to the point where they approximated the control. In addition, the application of curcumin alone had no significant difference in these parameters compared to the control and curcumin+sodium arsenite groups. However, we observed no significant differences in the body and the testis weight as well as the DNA integrity and histone-protamine replacement in the spermatozoa of the four groups.

**Conclusion:**

Curcumin compensated for the toxic effects of sodium arsenite on a number of sperm parameters in adult mice.

## Introduction

Numerous evidence exists that today’s men have considerably lower sperm counts compared to those who lived 50 years ago (1). Aspects of male reproductive health may undergo changes and induce infertility in men such as alterations in sperm production, maturation and fertilizing ability. Male reproductive abnormalities may be attributed to exposure to environmental toxicants such as arsenic (2). The toxic metalloid arsenic is released into the environment through industrial activities such as smelting of metals as well as coal burning (3) which contaminates drinking water (4). In addition, arsenic is used in food preservatives, herbicides, and insecticides (3). Drugs also contain arsenic (5). Therefore humans are exposed to this toxicant via different ways. Arsenic can exert adverse effects on the male reproductive system by altering reproductive hormones (6), sperm parameters (2,6), testicular enzymes, and testis histopathology (2,7). An increasing body of evidence suggests that oxidative stress is a possible mechanism in which arsenic damages organs (8,9). In this context, arsenic generates reactive oxygen species (ROS) which affects testicular function (8). In acute conditions, this will result in male infertility. 

Oxidative stress is associated with risk factors for infertility. Therefore, numerous studies have attempted to ameliorate the adverse effects of oxidative stress using antioxidant therapy to improve the endogenous antioxidant defense system. In this regard, application of antioxidants derived from plants can be an effective strategy for alleviating infertility caused by oxidative stress. Curcumin, a yellow phenolic pigment and active component of the rhizomes of Curcuma longa (turmeric), has a wide range of pharmacological and biological properties that include anti-inflammatory and anticancer effects (10,12). Curcumin is an antioxidant (13,14) that protects against oxidative damage in lipids (15), proteins (16), and DNA (17). The antioxidant property of curcumin is proposed to be related to its phenolic hydroxyl and methoxyl group on the phenyl ring. The phenolic hydroxyl group is located at the ortho position with respect to the methoxy group, which substantially increases the antioxidant activities of curcumin (18). Curcumin is reported to protect the male reproductive tract against the damaging effects of lipid peroxidation induced by oxidative stress (19,21). 

Previous studies reported adverse effects of sodium arsenite on the male reproductive tract (2,6). 

To our knowledge, however, no study examined the effects of curcumin on sodium arsenite mediated toxicity in epididymal spermatozoa of adult mice. The present study investigated the effects of curcumin on epididimal sperm parameters in adult mice exposed to sodium arsenite. 

## Materials and Methods

### Animals and exposures

In this experimental study, adult male Navel Medical Research Institute (NMRI) mice (8-9 weeks old, 32 ± 5 g) were purchased from Pasteur Institute, Tehran, Iran. The animals were housed in plastic cages on a 12-hour light/dark cycle, temperature of 24 ± 2ºC, with water and food ad *libitum*. Adult mice (n=24) were randomly divided into four groups: control, sodium arsenite (5 mg/kg, Sigma, USA) (2,6), curcumin (100 mg/kg, Sigma, USA) (22,23) and curcumin+sodium arsenite. Exposures were performed by intraperitoneal injections for a period of five weeks (one spermatogenic cycle for mice) (24). The local Ethical Committee at Arak University approved the experiments. Sodium arsenite and curcumin were dissolved in distilled water and dimethyl sulfoxide (DMSO, Merck, Germany), respectively. Based on the solvents, we chose two control groups, distilled water and DMSO. Since there were no significant differences between the results of the control groups, we considered data from the distilled water group as the control group. At the end of the exposures, the animals were weighed, anesthetized and dissected. Their left testes and cauda epididymides were removed. We recorded the testes weights. 

### Sperm count

The dissected epididymis from each animal was transferred into 10 ml Ham’s F10 medium and cut into small slices in order to release the spermatozoa into the medium. After 10 minutes, one ml of the sperm suspension was diluted with 9 ml of formaldehyde. The diluted spermatozoa were transferred into a Neubauer hemocytometer chamber and the sperm heads were counted with a microscope. The sperm count was performed according to the World Health Organization (WHO) guidelines (25) and data were expressed as the number of sperm per ml. 

### Sperm motility

Assessment of sperm motility was performed according to a WHO protocol (25). Briefly, 10 μl of the sperm suspension was placed on a prewarmed Mackler chamber. A minimum of 5 microscopic fields were assessed to evaluate sperm motility of at least 200 sperm for each of the animals. The percentage of sperm motility was analyzed for the following motion patterns: progressively motile sperm (PMS), nonprogressively motile sperm (NPMS), and nonmotile sperm (NMS). 

### Sperm viability

Sperm viability was evaluated by eosin-nigrosin staining according to a WHO protocol (25). In brief, 40 µL of eosin stain (1% in distilled water, Merck, Germany) was mixed to 20 µL sperm suspension. After 30 seconds, we added 60 µL of nigrosin stain (10% in distilled water, Merck, Germany). One drop of the mixture was placed on a microscope slide to generate a thin smear and examined under a light microscope at ×1000 magnification. In this method, viable spermatozoa remained colorless while nonviable spermatozoa stained red. 

### Sperm morphology

The Diff Quick staining kit (Faradid Pardaz Pars Inc., Iran) was used to evaluate sperm morphology (26). The sperm suspension was smeared onto a slide and air-dried. These smears were subsequently fixed in Diff Quick fixative for 25 seconds. The smears were then stained with Diff Quick staining solutions I and II for 25 seconds, then washed in distilled water. We observed 100 spermatozoa in order to detect the presence of sperm abnormalities in each sample. In the Diff Quick smear, acrosome stained pink (light purple) whereas the nucleus, midpiece and tail of the sperm stained dark purple. 

### Acrosome integrity

The ability of spermatozoa to undergo acrosome reaction was evaluated by the Coomassie brilliant blue staining method (27). The sperm suspension was smeared and air-dried. The air-dried smears were fixed in 5% paraformaldehyde/phosphate-buffered saline (PBS) for 15 minutes, then washed with PBS. The smears were subsequently stained with Coomassie brilliant blue solution (0.25% in 10% glacial acetic acid and 25% methanol) for 5 minutes after which they were washed with PBS. We counted 100 spermatozoa in each sample under a light microscope. In this staining, reactive acrosomes remained colorless, whereas intact acrosomes stained blue. 

### Sperm chromatin structure

In order to investigate sperm chromatin structure in epididymal sperm, acridine orange (AO) staining was used to detect DNA integrity (double strand DNA versus single strand DNA) in the sperm. Aniline blue (AB) staining was performed to detect histone-protamine replacement during the sperm maturation process. AO staining was performed according to the Tejada et al. (28) method. In brief, the smears were fixed with methanol/acetic acid (3:1) for 14 hours at 4°C and stained with the AO solution (0.19% in phosphate citrate buffer, pH=2.5) for 10 minutes. The slides were washed in distilled water, air dried, and then observed under fluorescence microscope (Olympus, Japan, excitation: 450-490 nm) at ×1000 magnification. At least 100 spermatozoa per slide were counted to evaluate double-strand DNA (green fluorescent) and single-strand DNA (yellow/red fluorescent). As a positive control, sperm DNA was denatured by heating at 96°C for 30 minutes in a thermocycler prior to staining. 

The AB staining was carried out based on the Wong et al. (29) method. The smears were immersed in 4% formalin (Merck, Germany) solution for 5 minutes. Fixed smears were then washed with distilled water and dipped in 5% AB stain in 4% acetic acid (pH=3.5) solution for 5 minutes. The slides were then washed in distilled water and stained with 0.5% eosin for 1 minute. Finally, we evaluated 100 spermatozoa per slide under a light microscope at ×1000 magnification. Spermatozoa were classified as dark blue (immature sperm with histone) and red-pink (mature sperm with protamine). As a positive control, sperm samples obtained from testis of immature mice were stained as previously mentioned. 

### Statistical analysis

Results were expressed as mean ± SD for six animals per group. One-way analysis of variance (ANOVA) followed by the Tukey’s test was used to assess the statistical significance of data using SPSS (SPSS for Windows, Version 16.0., Chicago, SPSS Inc.) P<0.05 was considered significant. 

### Results

We observed no significant differences in body and testis weights among the four groups (P>0.05, Table 1). 

**Table 1:** Absolute body and testis weights of male study mice


Weight (g)	Control	Curcumin	Sodium arsenite	Curcumin+sodium arsenite

Body	34.57 ± 2.17^a^	34.08 ± 3.34^a^	33.75 ± 4.32^a^	33.87 ± 1.32^a^
Testis	0.115 ± 0.01^a^	0.111 ± 0.03^a^	0.107 ± 0.01^a^	0.108 ± 0.01^a^


Mean ± SD, n=6 per group. P>0.05. Means with the same superscripts do not differ significantly. P>0.05. ANOVA, Tukeyʼs test.

There was significantly lower sperm count in the sodium arsenite group compared to the control group (P<0.01). In the curcumin+sodium arsenite group, curcumin significantly compensated the sperm count compared to the sodium arsenite group (P<0.01). Animals exposed with curcumin alone showed no significant difference in this parameter compared to the control and curcumin+sodium arsenite groups (Table 2). 

Sodium arsenite significantly decreased the percentage of PMS (P<0.001) and increased the percentage of NPMS (P<0.01) as well as NMS (P<0.001) compared to the control group. In the group of animals exposed to curcumin+sodium arsenite, curcumine significantly (P<0.001) ameliorated the adverse effect of sodium arsenite on these motility patterns compared to the sodium arsenite group. There were no significant differences in sperm motility in the curcumin group compared to the control and curcumin+sodium arsenite groups (Table 2). 

Sperm viability significantly (P<0.001) decreased in the sodium arsenite group compared to the control group. Administration of curcumin significantly reversed sperm viability in the curcumin+sodium arsenite group when compared to the sodium arsenite group (P<0.001). However, the application of curcumin alone had no significant effect on sperm viability compared to the control and curcumin+sodium arsenite groups (Table 2). 

**Fig.1:**
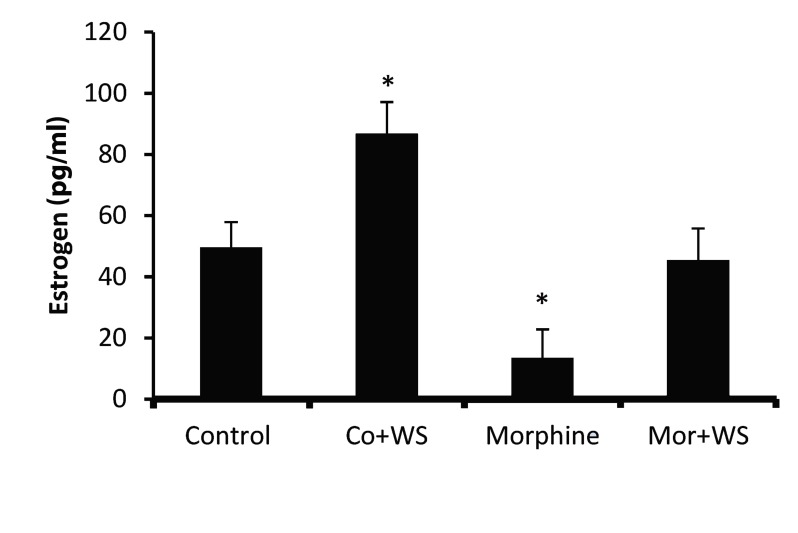
Sperm morphological abnormalities in sodium arsenite-exposed mice. A. Club-shaped head, B. Amorphous head, C. Bent head and D. Lack of the usual hook. Diff Quick staining (magnification: ×1000).

This table shows that the sodium arsenite induced a significant (P<0.01) increase in abnormal sperm. In the curcumin+sodium arsenite group, curcumin significantly reversed the percentage of sperm morphological abnormalities compared to the sodium arsenite group (P<0.01). Figure 1 shows a number of sperm abnormalities induced in sodium arsenite mice. Curcumin showed no significant effect on this parameter compared to the control and curcumin+sodium arsenite groups (Table 2). 

**Table 2:** Epididymal sperm number, sperm motility, sperm viability, sperm morphological abnormalities and acrosome integrity of male study mice


Sperm parameter	Control	Curcumin	Sodium arsenite	Curcumin+sodium arsenite

Number (10^6^)	9.53 ± 0.93^b^	9.69 ± 0.68^b^	5.55 ± 0.68^a^	9.02 ± 0.57^b^
PMS (%)	81.89 ± 1.29^b^	83.69 ± 1.90^b^	57.35 ± 1.52^a^	81.82 ± 0.91^b^
NPMS (%)	5.06 ± 0.71^b^	3.54 ± 0.90^b^	14.43 ± 1.42^a^	5.06 ± 0.46^b^
NMS (%)	13.05 ± 2.01^b^	12.77 ± 1.57^b^	28.22 ± 0.70^a^	13.12 ± 0.73^b^
Viability (%)	77.39 ± 5.68^b^	85.70 ± 3.74^b^	62.07 ± 7.59^a^	83.22 ± 2.70^b^
Morphological abnormalities (%)	13.32 ± 4.41^b^	11.15 ± 1.26^b^	24.90 ± 9.25^a^	13.36 ± 3.47^b^
Acrosome integrity (%)	74.96 ± 3.53^b^	78.86 ± 2.26^b^	60.56 ± 8.39^a^	75.99 ± 2.83^b^


PMS; Progressively motile sperm, NPMS; Non-PMS, and NMS; Non-motile sperm. Mean ± SD, n=6 per group. Means with the same super- scripts do not differ significantly. P<0.05. ANOVA, Tukey’s test.

In the sodium arsenite group there was a significantly lower percentage of spermatozoa with intact acrosome (P<0.001) compared to the control. Curcumin significantly (P<0.001) compensated the adverse effects of sodium arsenite on acrosomal reaction in the curcumin+sodium arsenite group compared to the sodium arsenite group. In addition, we observed no significant differencein acrosome integrity between the curcumin and control as well as curcumin+sodium arsenite groups (Table 2). 

Interestingly, in all sperm parameters where curcumin in the curcumin+sodium arsenite group significantly reversed the toxic effect of sodium arsenite, the data mean was similar to that of the control group (Table 2). 

**Fig.2:**
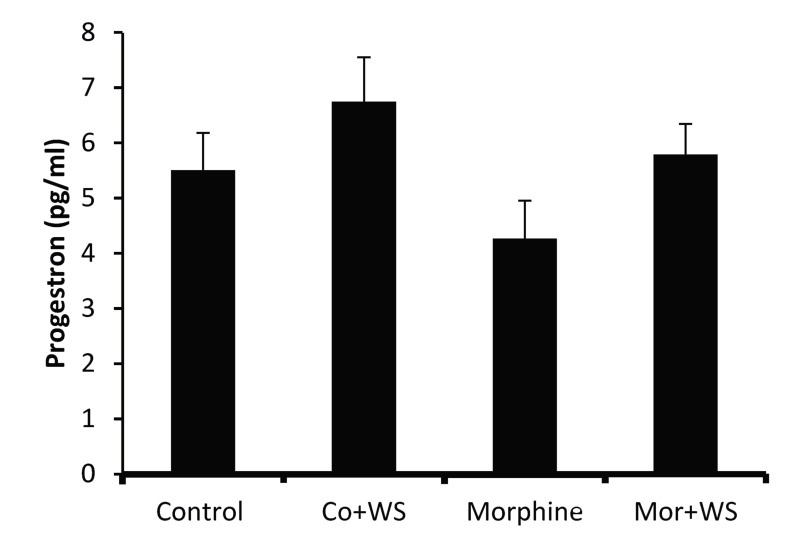
DNA integrity [acridine orange (AO) staining] and histone-protamine replacement [aniline blue (AB) staining] in mouse epididymal spermatozoa. AO staining: A. Control, B. Sodium arsenite (5 mg/kg), C. Positive control. AB staining: D. Control, E. Sodium arsenite (5 mg/ kg) and F. Positive control (magnification: ×1000).

Both AO and AB staining showed that neither sodium arsenite nor curcumin had any obvious effects on sperm DNA integrity and histone-protamine replacement (Fig .2,Table3). 

**Table 3:** DNA integrity and histone-protamine replacement in mouse epididymal sperm


Sperm parameter	Control	Curcumin	Sodium arsenite Curcumin+sodium arsenite

DNA integrity (%)	99.67 ± 0.52^a^	99.83 ± 0.41^a^	99.33 ± 0.82^a^	99.50 ± 0.84^a^
Histone-protamine replacement (%) 98.67 ± 1.03^a^	98.83 ± 0.75^a^	98.17 ± 1.17^a^	98.50 ± 1.04^a^


Mean ± SD, n = 6 per group. Means with the same superscripts do not differ significantly. P>0.05. ANOVA, Tukeyʼs test.

### Discussion

This study evaluated the spermatotoxic effects of sodium arsenite in adult mice and showed that curcumin had the capability to reverse sodium arsenite toxicity. 

In the present study, sodium arsenite had no significant effects on body and testis weights. Our results supported those of previous studies (7,30), however, other reports showed that arsenic exposure induced reductions in body and testis weights (31). These different results might be attributed to the dependency of the toxic effects of sodium arsenite on the dose and exposure period (32). 

In accordance with previous findings (2,6), sodium arsenite caused a significant reduction in the number of epididymal spermatozoa. The effect of sodium arsenite might have been attributed to the endocrine disrupting potential of arsenic (33). Therefore, the decreased sperm count might be the straight effect the reduction of luteinizing hormone (LH), follicle stimulating hormone (FSH), or testosterone production (6,34) which would reduce sperm counts in sodium arsenite-exposed mice. According to research, sodium arsenite induced apoptosis in the testis (35). Therefore, the reductions in sperm count might have been the result of sodium arsenite-mediated apoptosis in spermatogonia. In addition, arsenic is involved in the generation of free radicals (9). These indicators of oxidative stress can react with polyunsaturated fatty acids (PUFA), resulting in lipid peroxidation in the sperm membrane (36). Since curcumin is a potent antioxidant (10), it can reverse the adverse effects of sodium arsenite on sperm count. Therefore we have hypothesized that sodium arseniteinduced oxidative stress might be responsible for reductions in sperm count. 

The results of the present study showed a significant decrease in sperm motility and viability in the sodium arsenite group compared to the control group. Arsenic with its electrophilic nature has been shown to readily interact with thiol and sulfhydryl groups on proteins (37). The decline in the sperm motility might be due to the high concentration of arsenic in the epididymis where the sperm undergo the process of maturation and acquire motility. Oxidative stress mediated by sodium arsenite possibly damages cellular organelles such as mitochondria and in turn lead to disruption of mitochondrial membrane potential (38) and cellular ATP depletion (39). Therefore, we assumed that induction of oxidative stress by arsenic led to toxic effects on sperm motion kinetics and sperm viability. To support this idea we demonstrated that curcumin, with its antioxidant property, ameliorated the adverse effects of sodium arsenite on sperm motility and sperm viability parameters. 

This study has explored the toxic effect of sodium arsenite on sperm morphology. Arsenic can cause changes in pituitary-gonadal axis hormones (34). Therefore, it may be speculated that the appearance of sperm abnormalities are due to reductions in LH and FSH, with subsequent reduction in testosterone production. These hormonal alterations induced by arsenic may in turn affect spermatogenesis and reduce normal sperm morphology in the sodium arsenite-exposed mice. 

We showed that sodium arsenite significantly increased abnormal acrosome reaction in spermatozoa. Oxidative stress, by inducing lipid peroxidation, affected both fluidity and flexibility of spermatozoa membrane (40). The increase in the ability to undergo an abnormal acrosome reaction observed in animals exposed with sodium arsenite might have resulted from oxidative damage to the plasma membrane of spermatozoa. Acrosome reaction is a membrane fusion phenomenon which requires a high intracellular calcium concentration (41). Arsenic, by disrupting the endoplasmic reticulum, (38) can perturb calcium homeostasis to increase the cytoplasmic calcium concentration (42). Previous studies have shown that arsenic exposure caused a significant increase in calcium influx (43). Therefore, in the present study, sodium arsenite possibly increased cytosolic calcium in the spermatozoa, which led to a premature acrosome reaction. Compensation of this effect in the curcumin+sodium arsenite group might explain this possibility. 

Previous studies reported DNA damage induced by ROS (44). Antioxidant deprivation could cause DNA damage in spermatozoa (45). Furthermore, exposure of cells to arsenic induced DNA fragmentation (35). However, in this study sodium arsenite had no significant effect on epididymal sperm chromatin structure as evaluated by AO and AB staining. Several studies have shown that sodium arsenite exerts its effects in a doseand duration-dependent manner (32). It is reasonable to assume that this is the fact for these sperm parameters in the present study. In addition, mammalian sperm DNA has tightly compacted DNA compared to the other cells in the body (46). Tight packaging by protamines might protect sperm DNA against damaging agents. 

## Conclusion

Curcumin, as a major component of turmeric and a natural antioxidant, is not toxic. Its adverse effect on the fetus has not been reported. Possibly, curcumin can be used in human diets as a therapeutic agent against different pathological conditions induced by oxidative stress. 
